# Reversal of abnormal cardiac parameters following mitral valve replacement for severe mitral stenosis- in relation to pulmonary artery pressure-A retrospective study of noninvasive parameters

**DOI:** 10.1186/1749-8090-10-S1-A176

**Published:** 2015-12-16

**Authors:** Usha T Parvathy, Rajesh Rajan, Alexander Georgevich Faybushevich

**Affiliations:** 1 Department of Cardiac Surgery, Peoples Friendship University of Russia, Moscow, 118198, Russian Federation; 2 Department of Cardiology, Peoples Friendship University of Russia, Moscow, 118198, Russian Federation

## Background/Introduction

Though the regression of Pulmonary Arterial Hypertension (PAH) in mitral stenosis (MS) has been studied over varying periods post-intervention, corresponding studies on the cardiac chamber alterations after surgery are very limited.

## Aims/Objectives

We sought to determine the degree of reversal of these and the clinical status in connection with that of Pulmonary artery pressures (PAP).

## Method

The pre and one-year postoperative data;-{Functional Class (FC), cardiothoracic ratio (CTR) in chest x-ray, and echocardiographically Left atrium (LA), Right atrium (RA), Right ventricle (RV), left ventricle (LV), & Pulmonary Artery (PA) dimensions, PAP, tricuspid regurgitation (TR)}, of 50 patients who had Mitral valve replacement (MVR) for MS with PAH, were retrospectively analysed for correlations with PAP (Pearson's), and the change in each (t-test), in relation to that in PAP. PAH group-based (Group-I PAP ≥60 mmHg, Group-II >60 mmHg) analysis was done and the differences highlighted.

## Results

All parameters correlated with the baseline PAP, with statistical significance (p < 0.05), except LA (r = 0.081 p = 0.577). Postoperatively there was significant reduction in PAP, CTR, LA, RA, RV, PA, FC, TR (p < 0.001 each), and increase in LV (p < 0.003). The change in PAP was 39.42%; with the decrease in CTR, LA & RA related to it. The RV & PA showed lesser reduction (8.61 & 9.42%). The changes were greater & significant in Group-II (especially PAP, RV & PA). At one-year, PAP normalized in only 19 (38%). Residual PAH & chamber enlargement prevailed more in Gp-II.

## Discussion/Conclusion

Conclusions: This study emphasizes the importance of the baseline PAP in MS, to which was proportionate the functional disability & the cardiac chamber alterations (except LA). An improvement in these accompanied the postoperative PAP regression, though not all were directly proportionate with PAP. The higher-pressure group showed greater degree of regression, but also greater prevalence of residual abnormalities (most except LA), suggesting that the pathologic changes in them might take longer to resolve, which needs further evaluation.

